# Symptoms and their Relationship to Disability Following Treatment for Lower Extremity Tumours

**DOI:** 10.1080/13577149977677

**Published:** 1999-09

**Authors:** Aileen M. Davis, Sajeevan Punniyamoorthy, Anthony M. Griffin, Jay S. Wunder, Robert S. Bell

**Affiliations:** 1 The University Musculoskeletal Oncology Unit Mount Sinai Hospital and The University of Toronto Toronto Canada; 2 Suite 476H Mount Sinai Hospital 600 University Avenue Toronto M5G 1X5 Canada

## Abstract

*Purpose.* The aims of this study were to describe the symptoms experienced by patients in the first year following treatment for lower extremity sarcoma by limb conservation and to describe the relationship between symptoms and physical disability.

*Subjects.* Eighty consecutive patients treated for primary bone or soft tissue sarcoma (STS) of the lower limb who were treated with limb preservation surgery.

*Methods.* Subjects were evaluated by questionnaire at 6 weeks, and 3, 6, and 12 months post surgery. They identified whether they experienced any of the following symptoms: pain, stiffness, fatigue, weakness, limited range of motion, or swelling.The Toronto Extremity Salvage Score (TESS), a measure of physical disability, was also completed. Frequency of symptoms over time was calculated and change was evaluated using the Cochrane test. The relationship of symptoms to disability was analyzed with regression methods.

*Results.* The mean age was 43.0, SD=20.4 with a gender ratio of 1:1. There were 38 bone tumours and 42 STS.The most frequently reported symptoms were: stiffness 48 (60%), weakness 41 (51%), fatigue 26 (33%), and pain 25 (31%) at 6 weeks. Stiffness and fatigue decreased and plateaued by 3 months. Complaints of weakness and pain continued to decrease over time. At 6 weeks, pain, stiffness, weakness and limited motion predicted disability in both univariate and multivariate analyses. At 12 months, pain, stiffness, fatigue, weakness and limited motion were significant predictors of the TESS in univariate analysis with only pain, stiffness and limited motion significant predictors in the multivariate model.

*Discussion.* Pain, stiffness, fatigue, weakness and limited motion are common symptoms with stiffness and weakness decreasing significantly over time. The symptoms predictive of disability differ between the acute and late phases of recovery.


*Sarcoma* (1999) 3, 73–77

ORIGINAL ARTICLE

### Symptoms and their relationship to disability following treatment for lower extremity tumours

AILEEN M. DAVIS, SAJEEVAN PUNNIYAMOORTHY, ANTHONY M. GRIFFIN, JAY S. WUNDER & ROBERT S. BELL

*The University Musculoskeletal Oncology Unit, Mount Sinai Hospital and The University of Toronto, Toronto, Canada*

**Abstract**

*Purpose.* The aims of this study were to describe the symptoms experienced by patients in the first year following treatment for lower extremity sarcoma by limb conservation and to describe the relationship between symptoms and physical disability.

*Subjects.* Eighty consecutive patients treated for primary bone or soft tissue sarcoma (STS) of the lower limb who were treated with limb preservation surgery.

*Methods.* Subjects were evaluated by questionnaire at 6 weeks, and 3, 6, and 12 months post surgery. They identified whether they experienced any of the following symptoms: pain, stiffness, fatigue, weakness, limited range of motion, or swelling. The Toronto Extremity Salvage Score (TESS), a measure of physical disability, was also completed. Frequency of symptoms over time was calculated and change was evaluated using the Cochrane test. The relationship of symptoms to disability was analyzed with regression methods.

*Results.* The mean age was 43.0, SD=20.4 with a gender ratio of 1:1. There were 38 bone tumours and 42 STS. The most frequently reported symptoms were: stiffness 48 (60%), weakness 41 (51%), fatigue 26 (33%), and pain 25 (31%) at 6 weeks. Stiffness and fatigue decreased and plateaued by 3 months. Complaints of weakness and pain continued to decrease over time. At 6 weeks, pain, stiffness, weakness and limited motion predicted disability in both univariate and multivariate analyses. At 12 months, pain, stiffness, fatigue, weakness and limited motion were significant predictors of the TESS in univariate analysis with only pain, stiffness and limited motion significant predictors in the multivariate model.

*Discussion.* Pain, stiffness, fatigue, weakness and limited motion are common symptoms with stiffness and weakness decreasing significantly over time. The symptoms predictive of disability differ between the acute and late phases of recovery.

**Key words**: *lower extremity, tumours, disability, treatment*

### Introduction

Over the past two decades, limb conservation surgery for patients with extremity sarcoma has become the accepted standard of treatment. This standard has been attainable mainly due to the advent of modern imaging techniques and advances in surgical reconstructive techniques. The outcomes of patients treated by limb conservation have been reported in terms of survival and local control with few studies addressing the symptoms and disability experienced by patients following treatment.[1–4] Of these published studies reporting morbidity outcomes, many include patients treated in the 1970s and early 1980s, such that the outcomes related to treatment morbidity do not reflect current advances in treatment.[1–3] The aims of the current study were: to describe the symptoms experienced by patients in the first year following treatment by limb conservation for lower extremity sarcoma; to evaluate how the reported symptoms change over time; and to describe the relationship between symptoms and physical disability.

### Methods

From June 1994 to July 1995, 97 consecutive, consenting patients with primary bone or soft tissue (STS) sarcoma of the lower extremity were entered in the study. All patients were treated by limb conservation surgery. Use of adjuvant therapy, radiation or chemotherapy, was determined by the multidisciplinary team on a case by case basis. Adjuvant radiotherapy for soft tissue sarcoma was delivered as previously described by our group[5] and chemotherapy for patients with bone tumours varied according to the tumour histology. Patients presenting with metastatic disease, or who could not complete the questionnaires for reasons of language or cognition were excluded from the study.

Symptoms and the disability experienced by

Correspondence to: A. M. Davis, Suite 476H, Mount Sinai Hospital, 600 University Avenue, Toronto, Canada M5G 1X5., Tel: +1 416 586 8678; Fax: +1 416 586 8397.

1357-714X print/1369-1643 online/99/020073-05 © 1999 Taylor & Francis Ltd patients were collected by questionnaire at 6 weeks, and 3, 6 and 12 months post-operatively. Previous pilot interviews[6] identified that patients thought that pain, stiffness, fatigue, limited range of motion, weakness, swelling and abnormal sensation were important symptoms. Consequently, subjects were asked to indicate all symptoms that they experienced from the above list and they were also able to add additional symptoms. Physical disability was evaluated by the patient using the Toronto Extremity Salvage Score (TESS).[7,8] The TESS is a reliable and valid measure of physical disability developed for the extremity sarcoma population in which patients rate their ability to perform routine daily activities.[7]

Symptom data were analyzed by frequency at each point in time and change over time was evaluated graphically and using the Cochrane test. The relationship of symptoms to disability as measured by the TESS was evaluated in the immediate post-operative period (6 weeks) and then at 12 months post surgery when most patients had reached their maximal level of functioning. This relationship was evaluated using regression methods.

Of the 97 consecutive patients for whom functional outcome (TESS) data and symptoms data was available, only 80 had symptom data for all the time points. The data from these 80 subjects with complete data at all evaluation times formed the sample for analysis. The 17 subjects excluded from the analysis did not differ on any descriptive variables or their outcome from the sample of 80 (data not shown).

### Results

The subjects had a mean age of 43 years (± 20) and 41 were male. The sample included 38 subjects with primary bone tumours and the remaining 42 had primary soft tissue sarcomas. As anticipated in lower extremity musculoskeletal tumour patients, most tumours were proximal, specifically with 36 located in the area of the pelvis and hip, 35 at the knee and nine in the distal leg, foot and ankle. Of the bone tumour subjects, 11 were treated with adjuvant chemotherapy and 35 of the soft tissue sarcoma subjects received adjuvant radiotherapy.

At 6 weeks post surgery, pain (31%), stiffness (60%), fatigue (33%), weakness (51%) and limited range of motion (29%), were common symptoms experienced by patients (Table 1). With the exception of complaints of pain and swelling, the frequency of these symptoms decreased as the interval of time following surgery increased. Complaints of pain were relatively constant in frequency across patients until 6 months post surgery and then declined. Swelling was present in approximately 10% of cases at a given point in time. While appearing constant in frequency across time, the numbers actually reflect different subjects reporting swelling at a single point in time for half the cases. For instance, only a small number of cases ($n$=8), showed signs of swelling 6 weeks after surgery, and of these, less than half ($n$=3) showed signs of swelling at other time points. Hence, the number of subjects with persistent swelling across time was very small (3 of 80). Of particular note is the increasing frequency of subjects who have no symptom complaints in the year post surgery. By 12 months post-operatively, approximately 25% of subjects report no symptoms limiting their daily activities.

Considering that patients may not experience a single symptom in isolation, the data were analyzed to determine whether there were consistent symptom complexes reported. Specifically, we *a priori* evaluated the frequencies of three combinants of symptoms: (1) stiffness and weakness; (2) pain, stiffness and weakness; and (3) stiffness and limited range of motion. However, there did not appear to be consistent symptom complexes as the frequencies were 5% or less at all times for this patient sample.

The change in symptoms over time is shown graphically in Fig. 1a,b. There appear to be two patterns of symptom variation over time following treatment for lower extremity tumours. First, as shown in Fig. 1a, stiffness, fatigue and limited ROM show a plateau-type pattern. Stiffness is present to a great extent initially (60% of patients at 6 weeks) but then plateaus to approximately 45% of patients. Fatigue also plateaus after 3 months with approximately 23% ($n$=18) of patients subsequently experiencing this symptom. The data suggest that complaints of weakness and pain, however, do not plateau. Both pain and weakness are constant through 3 months and decline progressively through 6 and 12 months post surgery (Fig. 1b).

Statistical analysis of the change in frequency of symptoms over time using the Cochrane test and then McNemar's test *post hoc* to determine the specific

**Table 1**. *Frequency of symptoms in 80 subjects with lower extremity tumours treated by limb preservation*

| Symptom | 6 weeks (%) | 3 months (%) | 6 months (%) | 12 months (%) |
|---|---|---|---|---|
| No symptoms | 7 (9) | 12 (15) | 17 (21) | 21 (26) |
| Pain | 25 (31) | 28 (35) | 21 (26) | 16 (20) |
| Stiffness | 48 (60) | 37 (46) | 35 (44) | 34 (43) |
| Fatigue | 26 (33) | 17 (21) | 19 (24) | 19 (24) |
| Weakness | 41 (51) | 40 (50) | 29 (36) | 23 (28) |
| ROM | 23 (29) | 13 (16) | 13 (16) | 12 (15) |
| Swelling | 8 (10) | 5 (6) | 8 (10) | 6 (8) |

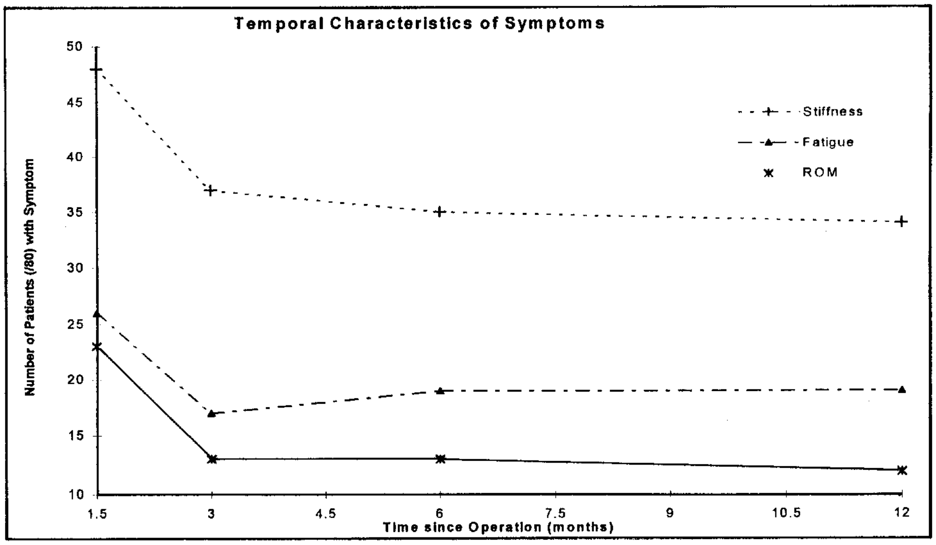

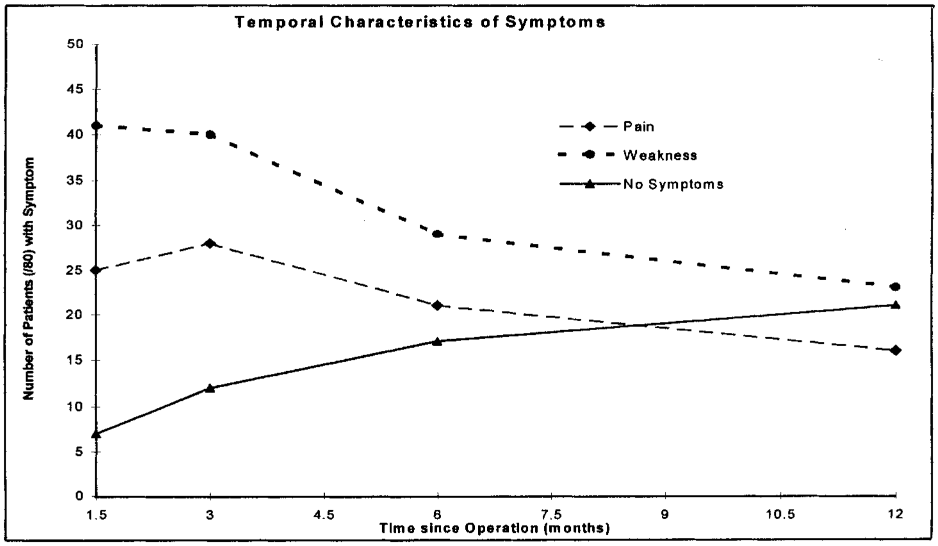

**Figure 1** *Frequency of symptoms over time following limb preservation surgery for lower extremity sarcoma. (a) Symptoms with a plateau in frequency over time. (b) Symptoms decreasing in frequency over time.*

time interval where change occurred confirms the data presented graphically. There is no statistically significant difference ($p$>0.05) in the frequency of complaints of pain, fatigue or swelling over time. There was a trend towards decreasing stiffness from 6 weeks post surgery to 6 months ($p$=0.04). There was also a trend towards decreasing complaints of weakness from 3 to 6 months ($p$=0.04), with a more significant decrease in complaints of weakness by 12 months from any of the earlier times ($p$ values ranging from 0.004 to 0.002). Decreasing complaints of limitation of range of motion were significant for all times compared to 6 week ($p$-values ranging from 0.03 to 0.01).

Physical disability was evaluated by the TESS. At 6 weeks post surgery, the mean TESS score was 64.3 (SD=23.9). Correlating the presence of each of pain, stiffness, fatigue, weakness, limited ROM or swelling with the TESS score, the correlation coefficients ranged from −0.31 to −0.39 (Spearman's ρ) indicating that the presence of the specific symptom resulted in lower TESS scores. All correlations were significantly different from zero. At 12 months, the mean TESS score was 83.9 (SD=15.8). Again the presence or absence of each symptom was correlated with the TESS score with the correlation coefficients ranging from −0.39 to −0.51. All correlations were significantly different than zero.

Regression analysis controlling for whether the patient had a bone or soft tissue sarcoma was used to evaluate the relationship of symptoms to physical disability as measured by the TESS. Table 2 shows that, at 6 weeks, complaints of pain, stiffness, weakness and limited ROM are significant predictors of disability in both univariate and multivariate analyses. In multivariate analysis, these symptoms explained 40% of the variance in the TESS score after controlling for whether the subject was treated for a bone or soft tissue tumour. At 12 months post surgery, pain, stiffness, fatigue, weakness, limited ROM were significant predictors of the TESS in univariate analysis. However, fatigue and weakness lost their

**Table 2.** *Symptoms as a predictor of physical disability as measured by the TESS*

| | P-value 6 Weeks | | P-value 12 Months | |
|---|---|---|---|---|
| Symptom | Univariate | Multivariate | Univariate | Multivariate |
| Pain | 0.000 | 0.028 | 0.000 | 0.001 |
| Stiffness | 0.000 | 0.018 | 0.000 | 0.000 |
| Fatigue | N/S | N/S | 0.000 | N/S |
| Weakness | 0.000 | 0.028 | 0.000 | N/S |
| ROM | 0.004 | 0.019 | 0.000 | 0.000 |
| Swelling | N/S | N/S | N/S | N/S |
| $R^2$ (variation in TESS explained) | N/A | 0.40 | N/A | 0.51 |

significance in the multivariate model. After controlling for bone versus soft tissue tumour, pain, stiffness, and limited ROM explained 51% of the variation in the TESS score of patients.

### Discussion

This study has confirmed that symptoms are an important outcome experienced by patients who undergo limb preservation surgery for extremity tumours. At least 80% of patients continue to experience some symptoms at 1 year post surgery. The symptoms most frequently reported include: stiffness (60%), weakness (51%), fatigue (33%), pain (31%), limited ROM (29%) and swelling (10%). These findings are similar to work of other authors who reported pain, swelling and weakness in soft tissue sarcoma patients[1–4] and pain and weakness in bone tumour patients.

To our knowledge, the current study is the first to evaluate how symptoms change over time. Two patterns were identified, one that shows that stiffness, fatigue and limited ROM remain quite constant after 3 months and another that suggests that pain and weakness continue to decrease in frequency 6 and 12 months post treatment. These patterns reflect tissue healing physiology. Soft tissue healing by scarring occurs in the first 3 to 6 weeks under normal conditions, but healing is prolonged in the face of radiotherapy and chemotherapy. Consequently, the first 3 months following surgery is the period during which rehabilitation is directed towards improving soft tissue lengthening and gaining joint motion by breaking soft tissue adhesions. Although strengthening is important in the early phases of rehabilitation, the most significant gains are observed later when strengthening can occur through the functional range of a joint. Furthermore, strengthening is more functionally based in the later stages of recovery as activity and endurance are emphasized.

The persistence of pain and weakness from 6 weeks to 3 months may also result from patients attempting more activities as they move further from their surgery.

Similarly, patients may report feeling weaker due to increased exertion. It is possible that patients cannot perform as many physical tasks as they attempt to do and therefore report weakness.

As noted above, the plateau in stiffness and ROM can be explained based on an understanding of tissue healing. However, the plateau in complaints of fatigue from 3 months is somewhat surprising. Fatigue was experienced by 33% of subjects at 6 weeks and at 3, 6 and 12 months reduced to approximately 23%. We would have anticipated that fatigue would have decreased as patients moved further from their treatment and improved their endurance. However, work in other cancers suggests that ongoing fatigue is a phenomenon associated with the cancer diagnosis. Specifically, in breast cancer, numerous authors[9–12] have found that fatigue is a frequent and persistent symptom.

The relationship between the presence of symptoms and the level of patient reported physical disability suggests that these two phenomena are moderately related with correlations in the range of magnitude of 0.30–0.50. The presence of symptoms accounts for up to 50% of the variance in the disability scores reported by patients in this sample. Although the impact of symptoms on physical disability has not previously been reported in the sarcoma population, work by Rigby *et al.*[13] in arthritic populations and McCarthy *et al.*[14] with injured workers would support these findings.

This study is the first to evaluate the trajectory of symptoms experienced by patients who undergo limb preservation for lower extremity sarcoma and the first to attempt to understand the relationship of these symptoms to the ability of patients to continue to perform their daily activities. Previous authors[1–4] have reported symptoms experienced by patients at a single point in time but many of these studies do not reflect current surgical or adjuvant therapy techniques. The recent trend in evaluating patient outcomes as a measure of treatment effectiveness has emphasized evaluation of functional status and quality of life. It is important that we recognize the component outcomes that reflect patient's status and how these outcomes relate to each other in order that new treatment interventions can be rigorously evaluated.

### Acknowledgement

This work was supported in part by funding from the National Cancer Institute of Canada—Clinical Trials Group.

### References

1 Lampert MH, Gerber LH, Glatstein E, *et al.* Soft tissue sarcoma: functional outcome after wide local excision and radiation therapy. *Arch Phys Med Rehabil* 1984; 65:477–80.
2 Stinson SF, Delaney TF, Greenberg J, *et al.* Acute and long-term effects on limb function of combined modality limb sparing therapy for extremity soft tissue sarcoma. *Int J Radiat Oncol Biol Phys* 1991; 21(6):1493–9.
3 Wexler AM, Eilber FR, Miller TA. Therapeutic and functional results of limb salvage to treat sarcomas of the forearm and hand. *J Hand Surg* 1988; 13a(2):292–5.
4 Robinson MH, Spruce L, Eeles R, *et al.* Limb function following conservation treatment of adult soft tissue sarcoma. *Eur J Cancer* 1991; 27(12):1567–74.
5 Wilson AN, Davis AM, Bell RS, *et al.* Local control of soft tissue sarcoma of the extremity: the experience of a multidisciplinary sarcoma group with definitive surgery and radiotherapy. *Eur J Cancer* 1994; 30A(6):746–50.
6 Davis AM. Limb Salvage Procedures for Bone and Soft Tissue Sarcomas: development of the measure of physical function. *Master of Science Thesis*. University of Toronto, 1994.
7 Davis AM, Wright JG, Williams JL, *et al.* Development of measure of physical function for patients with bone and soft tissue sarcoma. *Quality Life Res* 1996; 5(5):514–20.
8 Davis AM, Bell RS, Badley EM, *et al.* Evaluating functional outcome in lower extremity sarcoma patients: a comparison of four measures. *Clin Orthop* 1999; 358:90–100.
9 Cohen MZ, Kahn DL, Steeves, RH. Beyond body image: the experience of breast cancer. *Oncol Nurs Forum* 1998; 25(5):835–41.
10 Hann DM, Jacobsen PB, Azzarello LM, *et al.* Measurement of fatigue in cancer patients: development and validation of the fatigue symptom inventory. *Quality Life Res* 1998; 7(4):301–10.
11 Stein KD, Martin SC, Hann DM, Jacobsen PB. A multidimensional measure of fatigue for use with cancer patients. *Cancer Pract* 1998: 6(3): 143–52.
12 Wyatt GK, Friedman LL. Physical and psychosocial outcomes of midlife and olderwomen following surgery and adjuvant therapy for breast cancer. *Oncol Nurs Forum* 1998; 25(4):761–8.
13 Rigby AS, Rudofer SM, Badley EM, Brayshaw NC. The relationship between impairment and disability in arthritis: an application of the theory of generalized linear models to the ICIDH. *Int Disabil Stud* 1989; 11(2):84–8.
14 McCarthy ML, McAndrew MP, MacKenzie EJ, *et al.* Correlation between the measures of impairment, according to the modified system of the American Medical Association and function. *J Bone J Surg Am Version* 1998; 80A(7):1034–41.

## References

[PDF_00370] Cohen M. Z., Kahn D. L., Steeves R. H. (1998). Beyond body image: the experience of breast cancer.. Oncol Nurs Forum.

[PDF_00366] Davis A. M., Bell R. S., Badley E. M., Yoshida K., Williams J. I. (1999). Evaluating functional outcome in patients with lower extremity sarcoma.. Clin Orthop Relat Res.

[PDF_00373] Hann D. M., Jacobsen P. B., Azzarello L. M., Martin S. C., Curran S. L., Fields K. K., Greenberg H., Lyman G. (1998). Measurement of fatigue in cancer patients: development and validation of the Fatigue Symptom Inventory.. Qual Life Res.

[PDF_00339] Lampert M. H., Gerber L. H., Glatstein E., Rosenberg S. A., Danoff J. V. (1984). Soft tissue sarcoma: functional outcome after wide local excision and radiation therapy.. Arch Phys Med Rehabil.

[PDF_00389] McCarthy M. L., McAndrew M. P., MacKenzie E. J., Burgess A. R., Cushing B. M., Delateur B. J., Jurkovich G. J., Morris J. A., Swiontkowski M. F. (1998). Correlation between the measures of impairment, according to the modified system of the American Medical Association, and function.. J Bone Joint Surg Am.

[PDF_00384] Rigby A. S., Rudolfer S. M., Badley E. M., Brayshaw N. C. (1989). The relationship between impairment and disability in arthritis: an application of the theory of generalized linear models to the ICIDH.. Int Disabil Stud.

[PDF_00350] Robinson M. H., Spruce L., Eeles R., Fryatt I., Harmer C. L., Thomas J. M., Westbury G. (1991). Limb function following conservation treatment of adult soft tissue sarcoma.. Eur J Cancer.

[PDF_00377] Stein K. D., Martin S. C., Hann D. M., Jacobsen P. B. (1998). A multidimensional measure of fatigue for use with cancer patients.. Cancer Pract.

[PDF_00343] Stinson S. F., DeLaney T. F., Greenberg J., Yang J. C., Lampert M. H., Hicks J. E., Venzon D., White D. E., Rosenberg S. A., Glatstein E. J. (1991). Acute and long-term effects on limb function of combined modality limb sparing therapy for extremity soft tissue sarcoma.. Int J Radiat Oncol Biol Phys.

[PDF_00347] Wexler A. M., Eilber F. R., Miller T. A. (1988). Therapeutic and functional results of limb salvage to treat sarcomas of the forearm and hand.. J Hand Surg Am.

[PDF_00353] Wilson A. N., Davis A., Bell R. S., O'Sullivan B., Catton C., Madadi F., Kandel R., Fornasier V. L. (1994). Local control of soft tissue sarcoma of the extremity: the experience of a multidisciplinary sarcoma group with definitive surgery and radiotherapy.. Eur J Cancer.

[PDF_00380] Wyatt G. K., Friedman L. L. (1998). Physical and psychosocial outcomes of midlife and older women following surgery and adjuvant therapy for breast cancer.. Oncol Nurs Forum.

